# Antagonism of Cerebral High Mobility Group Box 1 Ameliorates Dendritic Cell Dysfunction in Sepsis

**DOI:** 10.3389/fphar.2021.665579

**Published:** 2021-08-26

**Authors:** Chao Ren, Ren-qi Yao, Li-xue Wang, Jun-cong Li, Kun-wei Chen, Yao Wu, Ning Dong, Yong-wen Feng, Yong-ming Yao

**Affiliations:** ^1^ Trauma Research Center, Fourth Medical Center and Medical Innovation Research Division of the Chinese PLA General Hospital, Beijing, China; ^2^ Department of Burn Surgery, Changhai Hospital, Naval Medical University, Shanghai, China; ^3^ Department of Critical Care Medicine, The Second People’s Hospital of Shenzhen, Shenzhen, China; ^4^ State Key Laboratory of Kidney Disease, The Chinese PLA General Hospital, Beijing, China

**Keywords:** high mobility group box-1 protein, sepsis, dendritic cells, cholinergic system, brain

## Abstract

Sepsis has emerged as a global health issue, and accounts for millions of deaths in intensive care units. Dysregulation of the immune response reportedly contributes to the pathogenesis and progression of this lethal condition, which involves both the dysfunction of immune cells and incompetent immunomodulatory mechanisms. High mobility group box 1 (HMGB1) is known as a later inflammatory mediator and is critically involved in the severity and prognosis of sepsis by inducing intractable inflammation and dysfunction of various immune cells. In the present study, we found that intracerebroventricular (ICV) injection of Box A, a specific antagonist of HMGB1, restored the dysregulated response of splenic dendritic cells (DCs) in septic mice by enhancing the expression of surface molecules, including CD80, CD86, and MHC-II, as well as improving DC priming of T lymphocytes. Cerebral HMGB1 was also confirmed to have potent inhibitory effects on DC functions when administrated by ICV injection in normal mice. The brain cholinergic system was found to mediate the immunomodulatory effects of central HMGB1, as it exhibited enhanced activity with persistent HMGB1 exposure. Furthermore, the inhibitory effects of cerebral HMGB1 on the response of peripheral DCs were also blocked by α7nAchR gene knockout. These findings provide novel insight into the relationship between cerebral HMGB1 and splenic DC dysfunction during sepsis, which is, at least in part, dependent on cholinergic system activity.

## Background

Sepsis has been recognized as a critical health issue worldwide and remains one of the leading causes of mortality in intensive care units. Dysregulation of the immune response reportedly contributes to the pathogenesis and progression of this lethal condition, according to the new definition of sepsis-3 (Rhodes et al., 2017). Abnormal immunological performance, including uncontrolled production of inflammatory mediators, dysfunctional immune cells, and dysregulated neuroendocrine immune networks, are commonly noted during sepsis (Hotchkiss et al., 2013). Re-establishing the functional homeostasis of the immune system might be benefit for septic patients. Dendritic cells (DCs) are the most important antigen-presenting cells and play an essential role in initiating the adaptive immune response. The impaired function of dendritic cell is critically involved in the development of sepsis-induced immunosuppression (Poehlmann et al., 2009; Bouras et al., 2018). However, the specific mechanism for sepsis-induced DC dysfunction remains unclarified.

Neuroendocrine immune networks have been confirmed with potent immunomodulatory properties and are associated with pathogenesis of multiple diseases. The neuroendocrine immune networks are composed of the following five parts: sensory nerve receptors, afferent pathways, cerebral nuclei, efferent nerves, and peripheral effective receptors. Abnormal response of neuroendocrine immune networks, either hyperactivation or hypoactivation, is reportedly detrimental to the responses of immune cells (Borovikova et al., 2000). For example, the cholinergic anti-inflammatory pathway (CAP) specializes in anti-inflammation, and has been well documented in various diseases after being identified by Tracey in 2000 (Borovikova et al., 2000). In the physiological state, cerebral cholinergic nuclei exhibit quick activation upon receiving inflammatory stimuli from both neuronal and humoral pathways, which further excites the vagus efferent nerve to induce an anti-inflammatory response and immunomodulation by releasing acetylcholine into the interspace between the axon terminal and immune cells expressing α7 nicotinic acetylcholine receptor (α7nAchR) (Pavlov and Tracey, 2006). α7nAchR has been identified as a unique peripheral receptor for the CAP and serves as a promising therapeutic target for inflammatory diseases (Ren et al., 2017a). Activation of the CAP by vagus nerve stimulation or α7nAchR agonists was shown to benefit septic animals by attenuating the excessive production of inflammatory cytokines and further improving survival rates, but these effects vanished by vagotomy or blocking α7nAchR (Wang et al., 2004; Pavlov et al., 2007; Kessler et al., 2012). In addition to controlling the inflammatory response, the cholinergic system negatively regulates the function of immune cells, such as suppressing the activation of macrophages and DCs, and augmenting the polarization of anti-inflammatory subtypes of T lymphocytes, which are mainly α7nAchR-dependent (Ren et al., 2017a). Inefficient or persistent activation of the cholinergic system, however, reportedly disrupts immune homeostasis. For example, α7nAchR-deficient mice showed more sensitivity to endotoxin insult than wild-type mice, along with uncontrolled production of inflammatory mediators (Wang et al., 2003). Augmented vagal activity was responsible for the immune paralysis during traumatic brain injury (Kox et al., 2008). It has been well documented that excessive production of inflammatory cytokine is a major sign of severe sepsis and responsible for multiple organ injury. The brain, for example, has been reported as the first organ subjected to inflammatory insult during septic exposure, and is even noted with irreversible damage if without prompt treatment (Gofton and Young, 2012). Uncontrolled production of proinflammatory cytokines is critically involved in brain injury by driving intractable neuroinflammation and massive neuronal apoptosis (Zhao et al., 2015). In septic animal models, increased level of tumor necrosis factor (TNF)-α directly resulted in cerebral edema and neuronal apoptosis, which were abrogated by TNFR1 deficiency (Alexander et al., 2008). Other proinflammatory mediators, such as interleukin (IL)-1β, IL-6, and high mobility group box-1 protein (HMGB1), have also been confirmed with great capacities in inducing neuroinflammation, followed by unfavorable impacts on the host immune response.

HMGB1 has been identified as a late proinflammatory cytokine that is released from activated monocytes or macrophages (Wang et al., 2014). Extracellular HMGB1 further induces activation and chemotaxis of immune cells, such as macrophages, neutrophils, and DCs, by binding with Toll-like receptor (TLR)2, TLR4, and the receptors for advanced glycation end products (RAGE) (Wang et al., 1999). Increased release of HMGB1 is considered with critical involvement in the severity of sepsis, and inhibition of HMGB1 significantly ameliorates multiple organ injury and improves the survival of septic animals (Karlsson et al., 2008; Barnay-Verdier et al., 2011). Furthermore, the DNA-binding domains of HMGB1 have been identified responsible for its proinflammatory activity, which mainly localizes to the Box B. While Box A, another DNA-binding domain of HMGB1, is capable of significantly inhibiting the activation of immune cells and release of proinflammatory cytokines by directly competing the binding sites between HMGB1 and associated receptors (Li et al., 2003). The use of Box A has been extensively accepted as an antagonist of HMGB1 and shown significant protection against multiple organ injury and sepsis lethality (Yang et al., 2004). We have previously identified that increased expression of cerebral HMGB1 was one of the major causes of sepsis induced brain injury, as shown by abnormal structure of brain tissues, massive neuronal apoptosis, and cognitive impairment, and these effects were alleviated by administration of BoxA (Ren et al., 2017b). However, the effects of HMGB1 on peripheral immune response and its relationship with the cholinergic anti-inflammatory pathway remain to be elucidated.

## Methods and Materials

### Animals

Wild type C57BL/6 mice (6–8 weeks, weighing 20 ± 2 g) were provided by the Laboratory Animal Science of the Chinese Academy of Medical Sciences, Beijing, China. Mice with α7nAchR gene knockout (α7nAchR-KO mice) were purchased from the Jackson Laboratory, Bar Harbor, Maine. These mice were housed in air-conditioned cages under a 12-h light and 12-h dark cycle with free access to water and food. All experiments were conducted in accordance with the National Institutes of Health (NIH) Guidelines, and approved by the Scientific Investigation Board of the Chinese PLA General Hospital (No. SYXK 2012-0014), Beijing, China.

### Catheter Insertion in Lateral Ventricles

The mice were placed on a motorized stereotactic apparatus (Stoelting Co., Wood Dale, IL) under anesthesia induced by isoflurane inhalation (induction: 3%; maintenance: 1.5%). The scalp was sterilized with 10% povidone-iodine after the hair was shaved. A 0.5-cm-long middle incision was made to expose the skull and visualize the bregma, which was further set as coordinate zero (x = 0, y = 0, z = 0). Then, a 0.5-cm diameter craniotomy was performed to insert the 2.5-cm-long catheter at the predetermined coordinates (x = −0.34 mm, y = −1.0 mm, z = 0). The incision was closed after the catheter was fixed with acrylic dental cement. The mice were allowed to rest and recover for at least 7 days before undergoing additional procedures.

### Cecal Ligation and Puncture Surgery

The septic mouse model was induced by CLP. The mice were placed on the operating table after anesthetization by an intraperitoneal injection of chloral hydrate (5%, 30 mg/kg of body weight). A 1-cm-long middle abdominal incision was made to expose the cecum after the skin was disinfected. The cecum was further ligated below the ileocecal valve and punctured once with a 21-gauge needle. A small amount of feces was extruded by slightly compressing the ligated cecum. Then, the incision was closed after relocating the cecum. All mice were given fluid resuscitation by subcutaneously injecting 1 ml of normal saline. Mice in the sham groups only underwent cecum exposure.

### Intracerebroventricular Injection

Mice were randomly divided into five groups: the sham CLP (sham-1) group, the sepsis group, the sham ICV injection (sham-2) group, the sepsis with ICV injection group (BoxA, 1 μg), and the HMGB1 injection group. In the sepsis with ICV injection group, BoxA solution (IBL international, Germany; 1 μg in 5 μl of normal saline) was injected into the lateral ventricle at 0 and 24 h after CLP. In the HMGB1 injection group, HMGB1 (Sigma-Aldrich, St. Louis, MO, 1 μg in 5 μl of normal saline) was injected at 0 and 24 h before the brain tissues and splenic DCs were harvested. ICV injection was performed in mice under isoflurane inhalation anesthesia. BoxA and HMGB1 were administered into the lateral ventricle through a preassembled syringe needle. The syringe was removed 5 min after injection to avoid backflow. Mice in the sham group were given the same volume of normal saline.

### Western Blotting

Tissues of different brain regions, including cortex and hippocampus, were collected, and homogenized in ice-cold RIPA lysis solution. The total protein in lysate was obtained after centrifuged (4°C, 12,000 rpm for 30 min) and further quantified by BCA kits. 75 μg protein samples were separated on 10% SDS/polyacrylamide gel. The protein was transferred to polyvinylidene fluoride membranes which were blocked by 10% nonfat milk and incubated with anti-mouse HMGB1 antibody (1:1,000) at 4°C over-night. The secondary antibody was incubated at room temperature for 1 h. The expression of HMGB1 was measured and analyzed by Investigator ProImage system.

### Isolation of Splenic DCs

Mouse splenocytes were obtained through a stainless steel mesh (70 μm) after the extraction and dispersion of the spleens. Mononuclear splenic cells were collected by Ficoll-Paque density gradient centrifugation (3,000 rpm, 15 min). CD11c^+^ DCs were then isolated from splenic mononuclear cells by a magnetic cell sorting system (Miltenyi Biotech, Bergisch Gladbach, Germany) according to the manufacturer’s instructions. Splenic mononuclear cells were incubated with CD11c microbeads (10 μl per 10^7^ cells) at 4°C for 15 min. Then, the CD11c^+^ DCs were collected after magnetic separation by MS columns.

### Phenotypic Analysis of DCs

DC phenotypes were analyzed by flow cytometry (BD Biosciences, Mountain View, CA) after the cells were incubated with the following antibodies: allophycocyanin (APC)-conjugated anti-mouse CD80, phycoerythrin (PE)-conjugated anti-mouse CD86, fluorescein isothiocyanate (FITC)-conjugated anti-mouse major histocompatibility complex (MHC)-II (Miltenyi Biotec GmbH, Bergisch Gladbach, Germany), FITC-conjugated anti-mouse CD206 and Brilliant Violet™ 421 (BV421)-conjugated anti-mouse CD163. DCs were resuspended in staining buffer and further incubated with the abovementioned antibodies at 4°C for 40–60 min. The expressions of CD80, CD86, MHC-II, CD206, and CD163 were measured by the flow cytometry.

### Isolation of Splenic T Lymphocytes and Cell Counting Kit-8 Assay

Splenic CD4^+^ T cells were isolated from splenic mononuclear cells *via* a magnetic cell sorting system in accordance with the manufacturer’s instructions. Splenic mononuclear cells were incubated with anti-mouse CD4 microbeads (20 μl/10^7^ cells) at 4°C for 15 min. CD4^+^ T cells were obtained after magnetic separation by MS columns. Purified CD4^+^ T lymphocytes were resuspended in complete RPMI 1640 medium (10% fetal calf serum, 100 U/ml penicillin, and 100 μg/ml streptomycin) and plated in 96-well plates at 4 × 10^5^ cells/well, after which the cells were cultured with soluble CD3 (1 μg/ml) and soluble CD28 (5 μg/ml) for 24 h. DCs were then added to the medium at a DC: T cell ratio of 1: 100, and cultured for 72 h. T lymphocytes proliferation was measured by a CCK8 kit (Dojindo Laboratories, Kumamoto, Japan) in accordance with the manufacturer’s instructions. Ten microliters of CCK8 solution were mixed with the cell suspension and further incubated for 2 h. The optical density value was recorded at 450 nm by an enzyme-linked immunosorbent assay (ELISA) plate reader (Spectra MR, Dynex, Richfield, MN).

### Measurements of Cytokine Levels

The levels of IL-2, IL-4, and interferon (IFN)-γ in the culture supernatants were quantified by ELISA kits (Excell Inc., Shanghai, China) according to the manufacturer’s protocols. The results were recorded and analyzed by an ELISA plate reader (Spectra MR, Dynex, Richfield, MN).

### Measurements of Acetylcholinesterase and Choline Acetyltransferase

Brain tissues, including cortex, hippocampus, and striatum, and splenic tissues were homogenized in PBS with a glass homogenizer. The suspensions were subjected to three freeze-thaw cycles and centrifuged (4°C, 12,000 rpm for 30 min) to collect the supernatants. The levels of AchE and ChAT were quantified by ELISA kits (MyBioSource Inc., San Diego, CA) and the ratios of these values to those in the sham groups were further analyzed. The serum was separated from peripheral blood that underwent centrifugation at 7,500 rpm for 10 min. The level of serum AchE was measured by ELISA kits, as mentioned above.

### Statistical Analysis

The data were analyzed by SPSS 19.0 software and are presented as the mean ± SD. One-way analysis of variance (ANOVA) was used to evaluate differences among multiple groups, and Student’s t-test was used to assess the significance of intergroup differences. *p* values less than 0.05 were considered significant.

## Results

### Antagonism of Cerebral HMGB1 Improved Sepsis Induced DC Dysfunction

**FIGURE 1:**
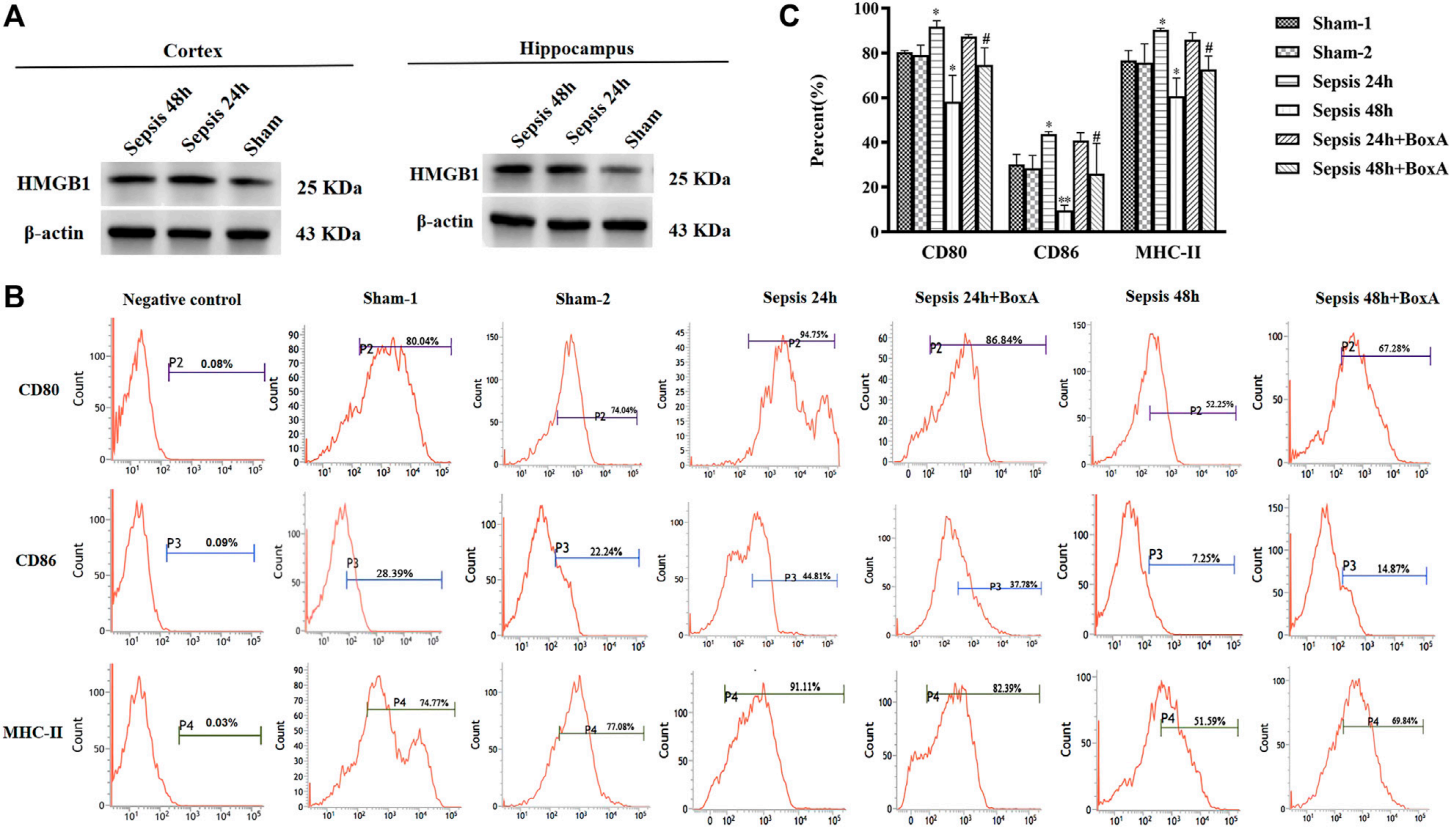
Antagonism of cerebral HMGB1 ameliorated immune dysfunction in splenic DCs following septic challenge. **(A)** The expression of HMGB1 protein in brain tissues, including cortex and hippocampus by western blotting. **(B)** The expression of costimulatory molecules in DCs, including CD80, CD86, and MHC-II, was measured by flow cytometry. **(C)** The statistical results are presented to indicate these phenotypic changes (sham-1: sham for the CLP surgery, *n* = 6; sham-2: sham for the intracerebroventricular (ICV) injection, *n* = 6; vs*.* the sham-1 group: ^*^ *p* < 0.05, **p < 0.01; vs*.* the sepsis group at the same time point: ^#^ *p* < 0.05).

Contents of brain HMGB1, including cortex and hippocampus, were significant increased after the induction of sepsis (Figure 1A). The expression of surface molecules on DCs was analyzed at 24 and 48 h after CLP surgery. As shown in Figures 1B,C, the expressions of CD80, CD86, and MHC-II were all increased at 24 h after the induction of sepsis. However, these molecules showed low expression in the sepsis 48 h group compared with both sham and sepsis 24 h groups. As reported in our previous study (Ren et al., 2017b), the release of cerebral HMGB1 was confirmed to have critical involvement in the development of sepsis-induced brain injury. To explore the effects of cerebral HMGB1 on the peripheral immune response, we measured the functional changes of splenic DCs after ICV injection of BoxA. The suppressed expression of CD80, CD86, and MHC-II was reversed by inhibiting cerebral HMGB1 at 48 h after sepsis. However, no significant differences were noted in the expression of costimulatory molecules between mice in the sepsis 24 h group and those in the sepsis 24 h group with ICV injection of BoxA.

**FIGURE 2:**
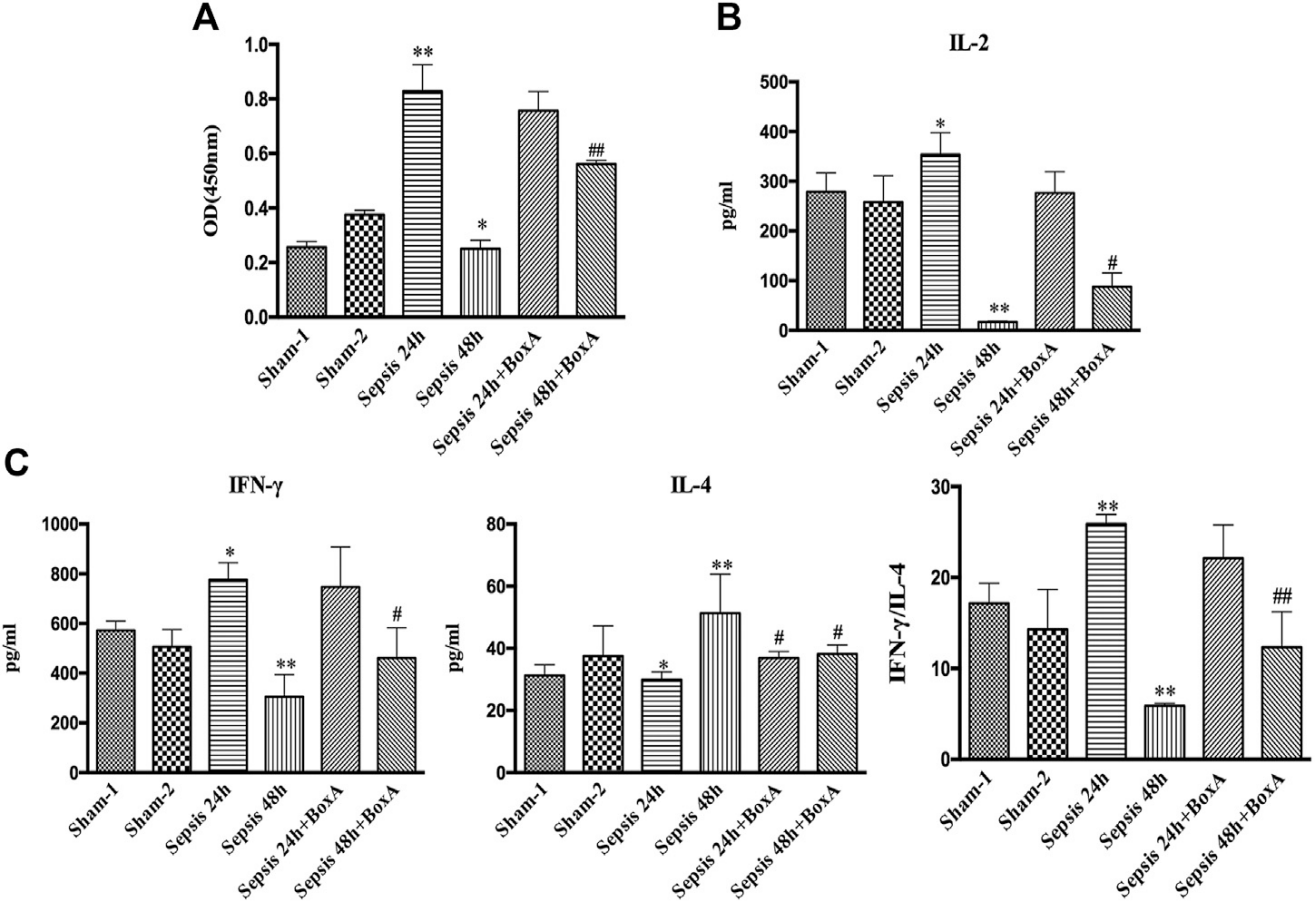
Antagonism of brain HMGB1 improved T lymphocyte priming by DCs o in sepsis. **(A)** The proliferation of CD4^+^ T cells was measured by the CCK8 assay and quantified by an ELISA plate reader at 450 nm. **(B, C)** The levels of IL-2, IFN-γ, and IL-4 in coculture supernatants were measured by ELISA kits, and the ratio of IFN-γ/IL-4 was determined to reflect the polarization of CD4^+^ T cells (*n* = 6, vs*.* the sham-1 group: ^*^ *p* < 0.05, **p < 0.01; vs*.* the sepsis group at the same time point: ^#^ *p* < 0.05, ^##^ *p* < 0.01).

We further measured the proliferation and cytokine secretion of T lymphocytes that were cocultured with DCs. The proliferation of T cell was increased at sepsis 24 h when compared with that in the sham group, while it was inhibited in the sepsis 48 h group, indicating ineffective DC priming under persistent exposure to sepsis (Figure 2A). IL-2 levels exhibited a similar trend, as evidenced by increased levels in the sepsis 24 h group but significantly decreased levels at 48 h after CLP (Figure 2B). The ratio of IFN-γ to IL-4 is a good marker of the polarization of T lymphocytes. In the sepsis 24 h group, the production of IFN-γ was increased compared with that in the sham group, while no significant changes were observed in IL-4 levels, followed by an elevated ratio of IFN-γ/IL-4, thus indicating the dominant differentiation of Th1 subtypes. However, in the sepsis 48 h group, we found lower levels of IFN-γ and higher production of IL-4 than those in both the sham and sepsis24 h groups, accompanied by a significantly suppressed IFN-γ/IL-4 ratio and polarization of Th2 subtypes (Figure 2C). Antagonism of cerebral HMGB1 improved the proliferation and IL-2 release of T cells and reversed the polarization of Th2 cells in the sepsis 48 h group. However, no significant differences were noted in the proliferation, IL-2 secretion, and IFN-γ/IL-4 ratio of T cells between sepsis 24 h group and sepsis 24 h with BoxA injection group.

**FIGURE 3:**
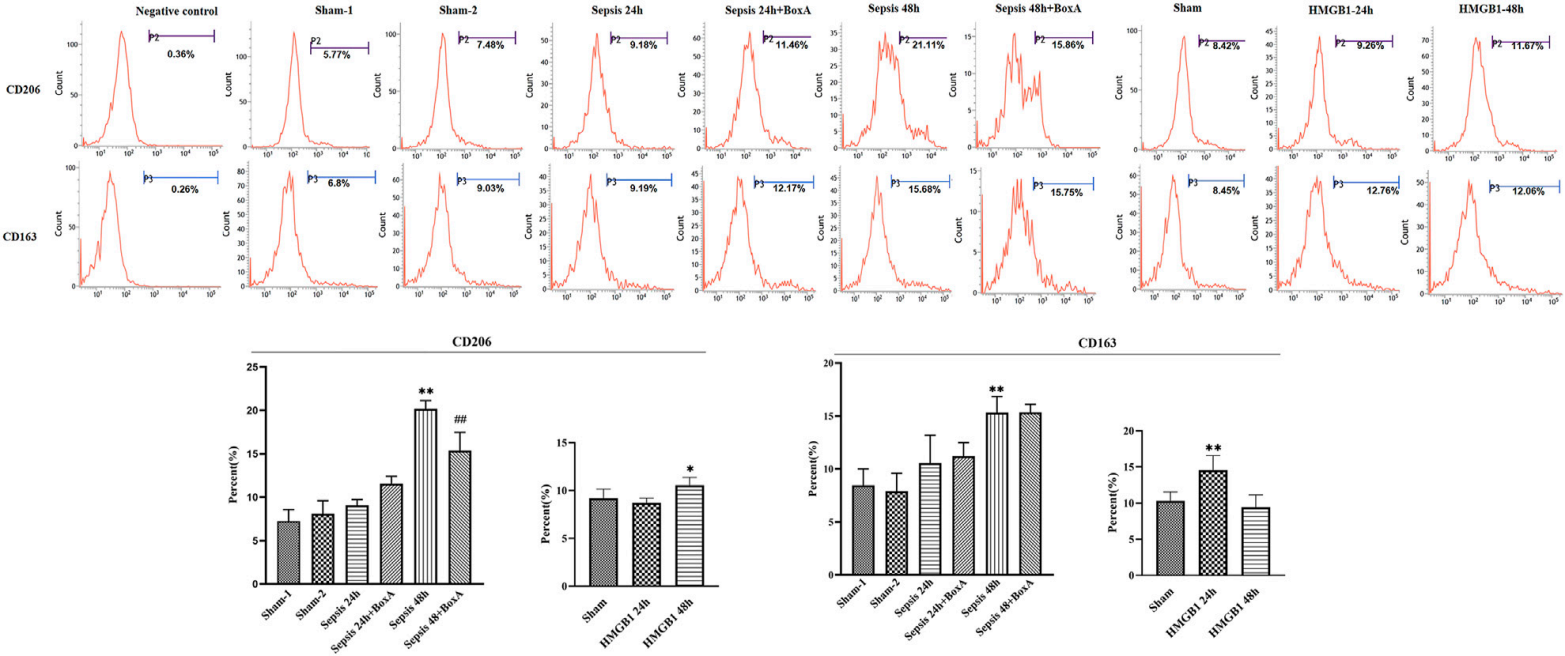
The effects of cerebral HMGB1 on the expressions of CD206 and CD163 in DCs. The expressions of CD206 and CD163 were determined by flow-cytometry. (*n* = 6; vs*.* the sham group: ^*^ *p* < 0.05, **p < 0.01; vs*.* the sepsis group at the same time point: ^# #^ *p* < 0.01).

Previous data showed the essential involvement of cerebral HMGB1 in sepsis-induced dysfunction of splenic DCs, as evidenced by inhibiting expressions of surface molecules and priming activity for T cells. We further evaluated the expressions of CD206 and CD163 and found that both molecules showed significantly upregulated expressions at 48 h post sepsis induction (Figure 3). The expression of CD206 was significantly downregulated by ICV injection of Box A when compared with that in the sepsis 48 h group. While no significant differences were noted in CD163 expressions between the sepsis 48 h group and the sepsis 48 h with Box A injection group. The expression of CD206 also showed significant increase after stimulated by brain HMGB1 for 48 h in comparison with that in the sham group. The upregulated CD163 expression was only noted at 24 h after ICV injection of HMGB1 when compared with that in the sham group.

**FIGURE 4:**
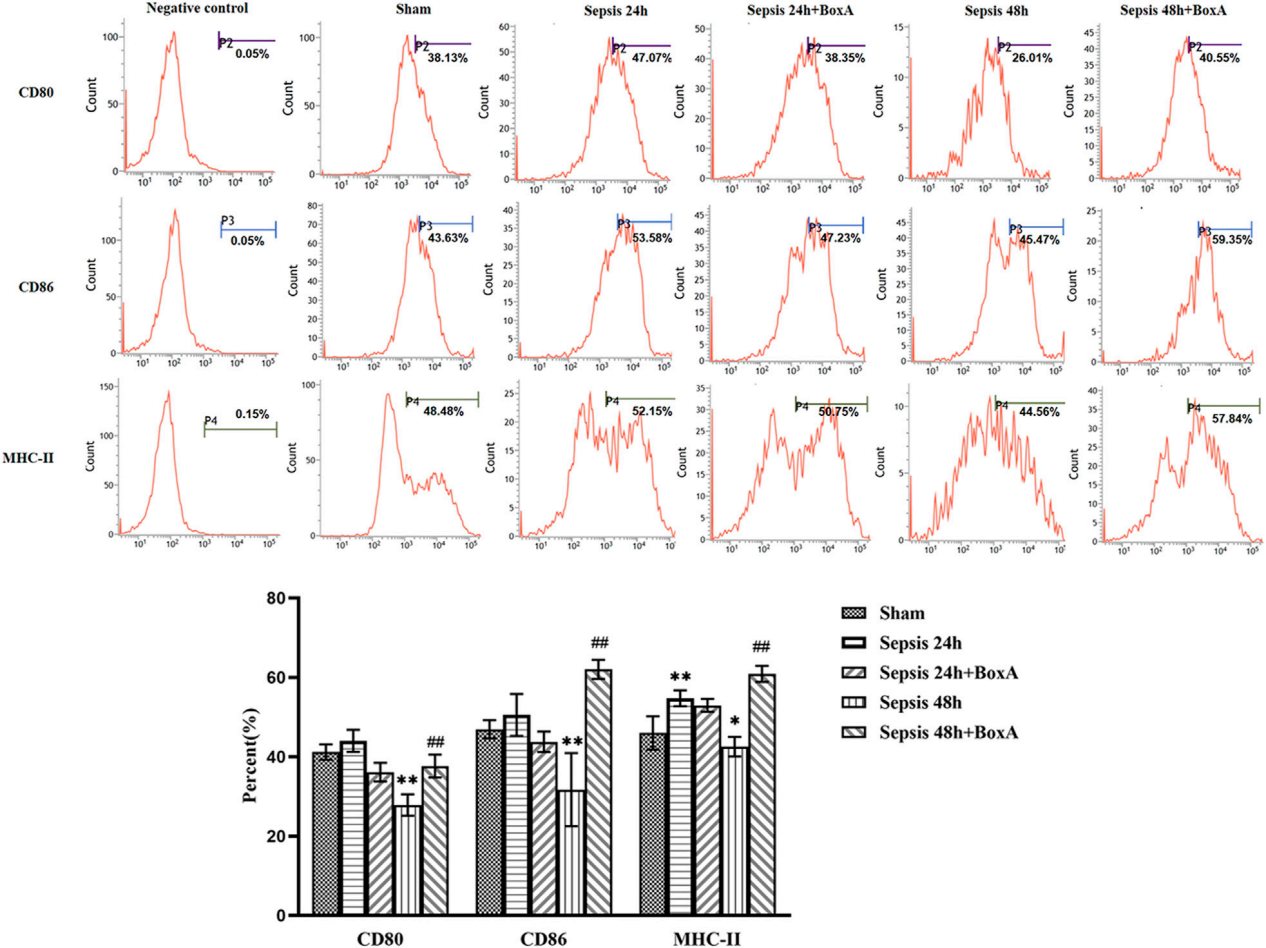
Intravenous use of Box A ameliorated immune dysfunction in splenic DCs following septic challenge. The expressions of costimulatory molecules on DCs, including CD80, CD86, and MHC-II, were measured by flow cytometry. The statistical results were presented to indicate these phenotypic changes (*n* = 6; vs*.* the sham group: ^*^ *p* < 0.05, **p < 0.01; vs*.* the sepsis group at the same time point: ^#^ *p* < 0.05).

The Box A has been identified with great protection against multiple organ injury and septic lethality by systematic use, i.e., intraperitoneal or intravenous. Herein, we evaluated the impacts of intravenous administration of Box A on the immune function of DCs. As shown in Figure 4, intravenous Box A significantly reversed the suppressive expression of surface markers, i.e., CD80, CD86, and MHC-II, at 48 h post septic challenge.

### Brain HMGB1 Induced DC Dysfunction by Triggering Hyperactivation of the Cholinergic System Following Septic Challenge

**FIGURE 5:**
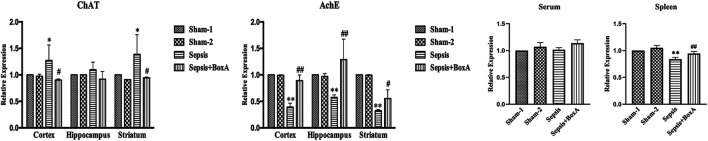
Inhibition of cerebral HMGB1 decreased the activity of the cholinergic system in septic mice. The levels of AchE and ChAT in tissues of different brain regions, including cortex, hippocampus, striatum, and serum as well as spleen were quantified by ELISA kits and analyzed as the ratios to those in the sham-1 groups (*n* = 6, vs*.* the sham-1 group: ^*^ *p* < 0.05, **p < 0.01; vs*.* the sepsis group at the same time point: ^#^ *p* < 0.05, ^##^ *p* < 0.01).

The activity of cholinergic system is critically involved in the pathogenesis and development of various diseases (Borovikova et al., 2000). We assessed the expressions of AchE and ChAT in different brain sections, including cortex, hippocampus, and striatum, at 48 h after CLP surgery. The levels of ChAT were increased in sepsis groups compared with those in sham groups, while AchE exhibited lower expression in the brain tissues of septic mice than those of sham mice. Inhibition of cerebral HMGB1 downregulated ChAT expression but resulted in increased expression of AchE, indicating that HMGB1 might be responsible for hyperactivation of the brain cholinergic system under sepsis (Figure 5). We further measured the levels of AchE in both serum and spleen and found that the expression of AchE was significantly decreased in the spleens of septic mice when compared with those in the sham-1 and sham-2 groups, which was reversed by ICV injection of BoxA. However, no significant differences were found in the level of AchE of serum samples.

### The Effects of Cerebral HMGB1 on the Immune Response of Splenic DCs

**FIGURE 6:**
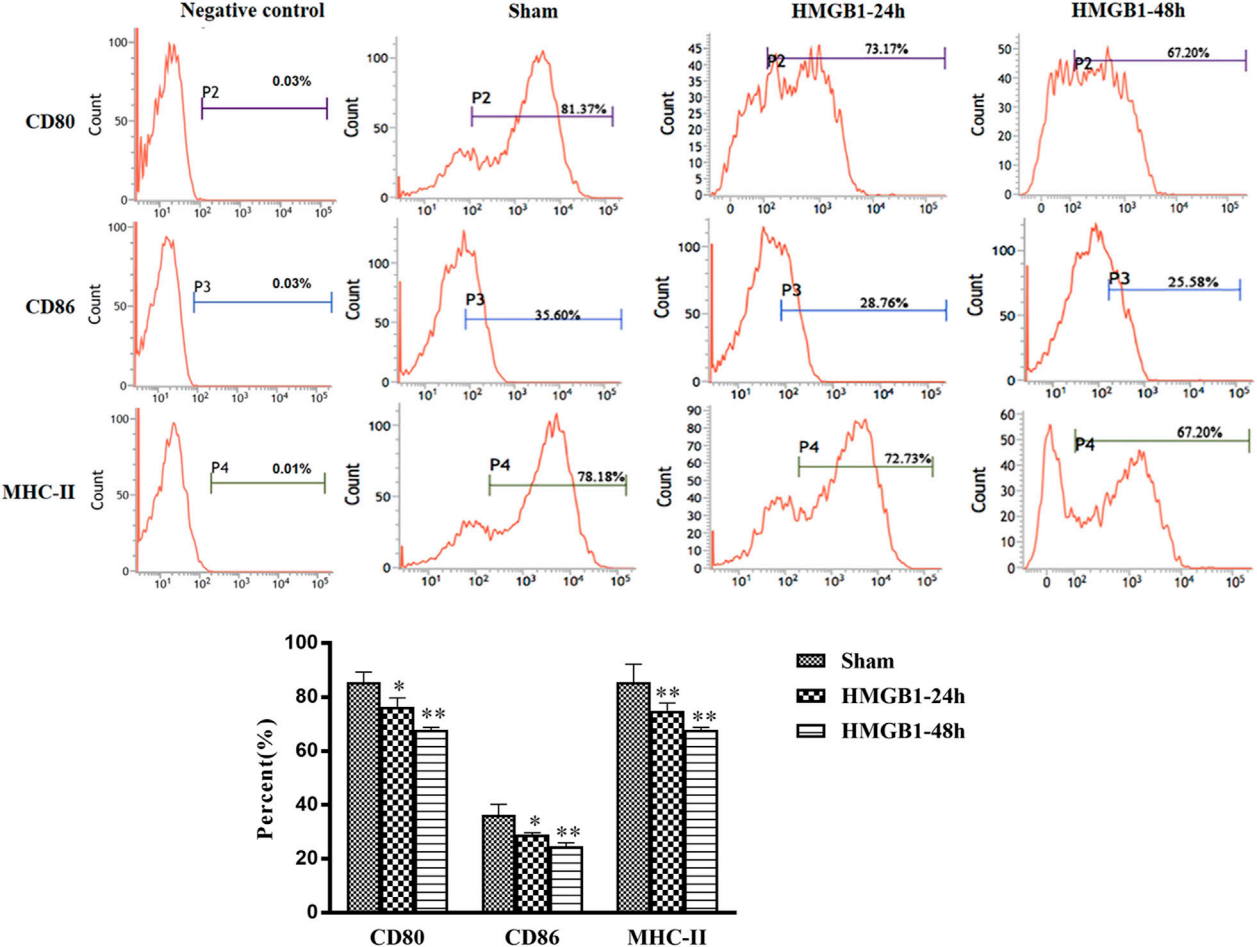
ICV injection of HMGB1 contributed to disturbed maturation and activation of splenic DCs in normal mice. The levels of CD80, CD86, and MHC-II were determined by flow cytometry. The statistical results are presented to indicate these phenotypic changes (*n* = 6, vs*.* the sham group: ^*^ *p* < 0.05, **p < 0.01).

**FIGURE 7:**
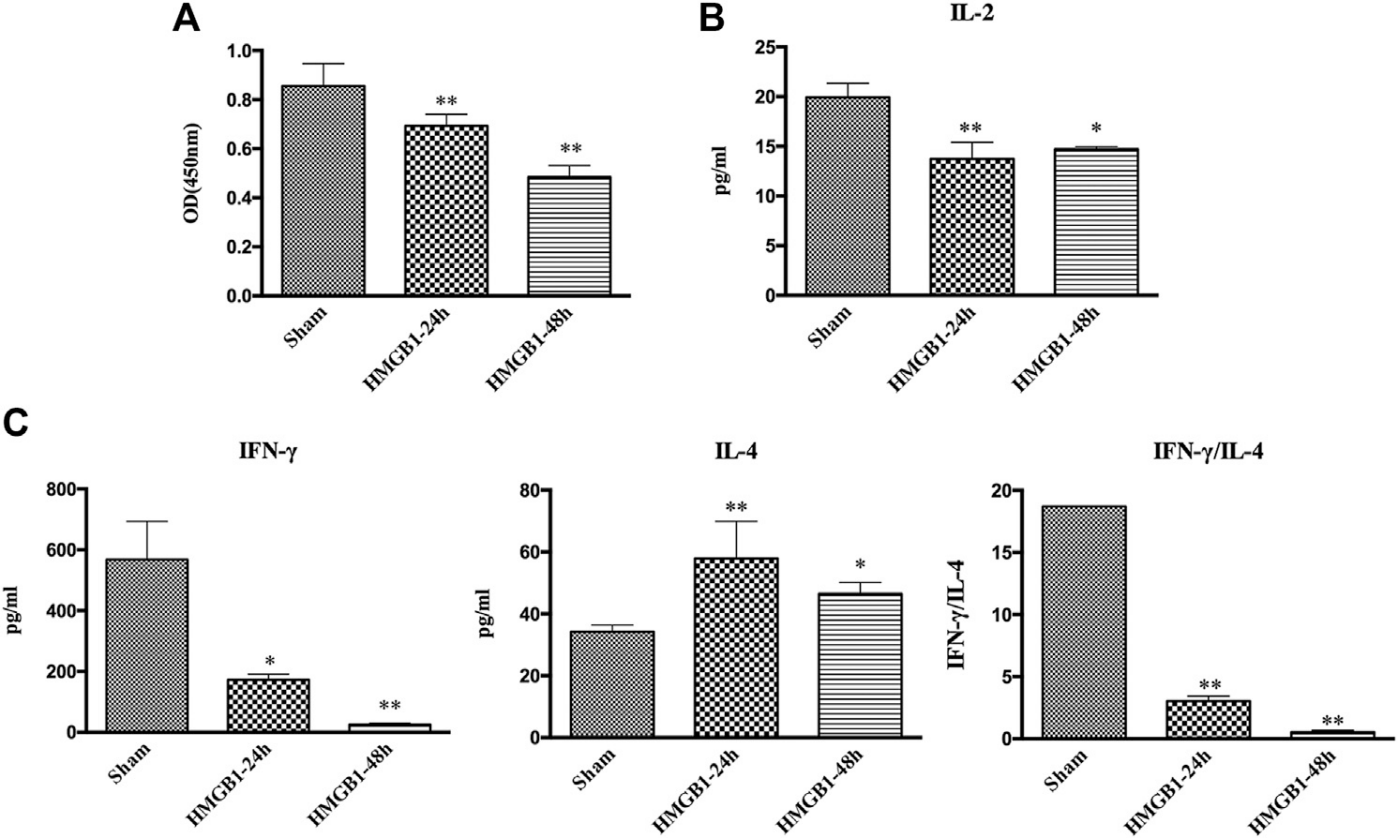
ICV injection of HMGB1 induced incompetent DC priming of T cells. **(A)** The proliferation of CD4^+^ T cells was measured by the CCK8 assay and quantified by an ELISA plate reader at 450 nm. **(B, C)** The levels of IL-2, IFN-γ, and IL-4 in coculture supernatants were determined by ELISA kits, and the ratio of IFN-γ/IL-4 was determined to reflect the polarization of CD4^+^ T cells (*n* = 6, vs*.* the sham group: ^*^ *p* < 0.05, **p < 0.01).

To clarify the impact of cerebral HMGB1 on the immune response of peripheral DCs, we conducted ICV injection of HMGB1 and found that the expression of surface molecules on DCs was significantly inhibited by ICV injection of HMGB1 in a time-dependent manner, as shown by decreased expression of CD80, CD86, and MHC-II when compared with those in sham group (Figure 6). Cerebral HMGB1 also disturbed the priming activity of DC for T cells. The proliferation and IL-2 secretion of T cells were suppressed after coculture with DCs that underwent stimulation by brain HMGB1 for 24 and 48 h when compared with those in the sham group (Figures 7A,B). The proliferative activity of T cells revealed more significant decrease at 48 h post brain HMGB1 stimulation. The differential profile indicated the polarization of Th2 subtypes, as the ratio of IFN-γ/IL-4 was lower in the HMGB1 24 and 48 h groups than those in the sham group, especially significant for 48 h after HMGB1 exposure (Figure 7C).

### Cerebral HMGB1 Resulted in Hyperactivation of the Brain Cholinergic System

**FIGURE 8:**
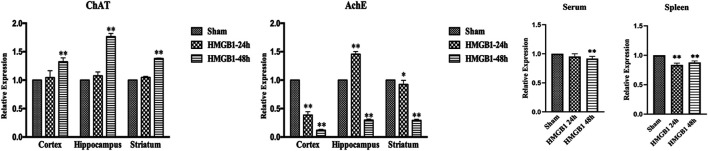
Cerebral HMGB1 enhanced the activity of the cholinergic system. The levels of AchE and ChAT in the tissues of different brain regions, including cortex, hippocampus, striatum, and serum as well as spleen were quantified by ELISA kits, and analyzed as the ratios to those in the sham groups (*n* = 6, vs*.* the sham group: ^*^ *p* < 0.05, **p < 0.01).

The activity of the brain cholinergic system was further evaluated after ICV injection of HMGB1. As shown in Figure 8, HMGB1 administration increased the expression of ChAT in the cortex, hippocampus, and striatum at 48 h after injection, but there was no significant increase at 24 h. The levels of brain AchE were notably reduced at 48 h after ICV injection of HMGB1. Levels of AchE were noted with significant decreases in both serum and spleens at 48 h after ICV injection of HMGB1 when compared with those in the sham groups. The expression of splenic AchE also showed obvious decrease at 24 h post cerebral HMGB1 stimulation, while no significant difference was noted in serum AchE between the sham group and the HMGB1 24 h group.

### Blockade of α7nAchR Reversed the Inhibitory Effects of Brain HMGB1 on the Immune Response of Splenic DCs

**FIGURE 9:**
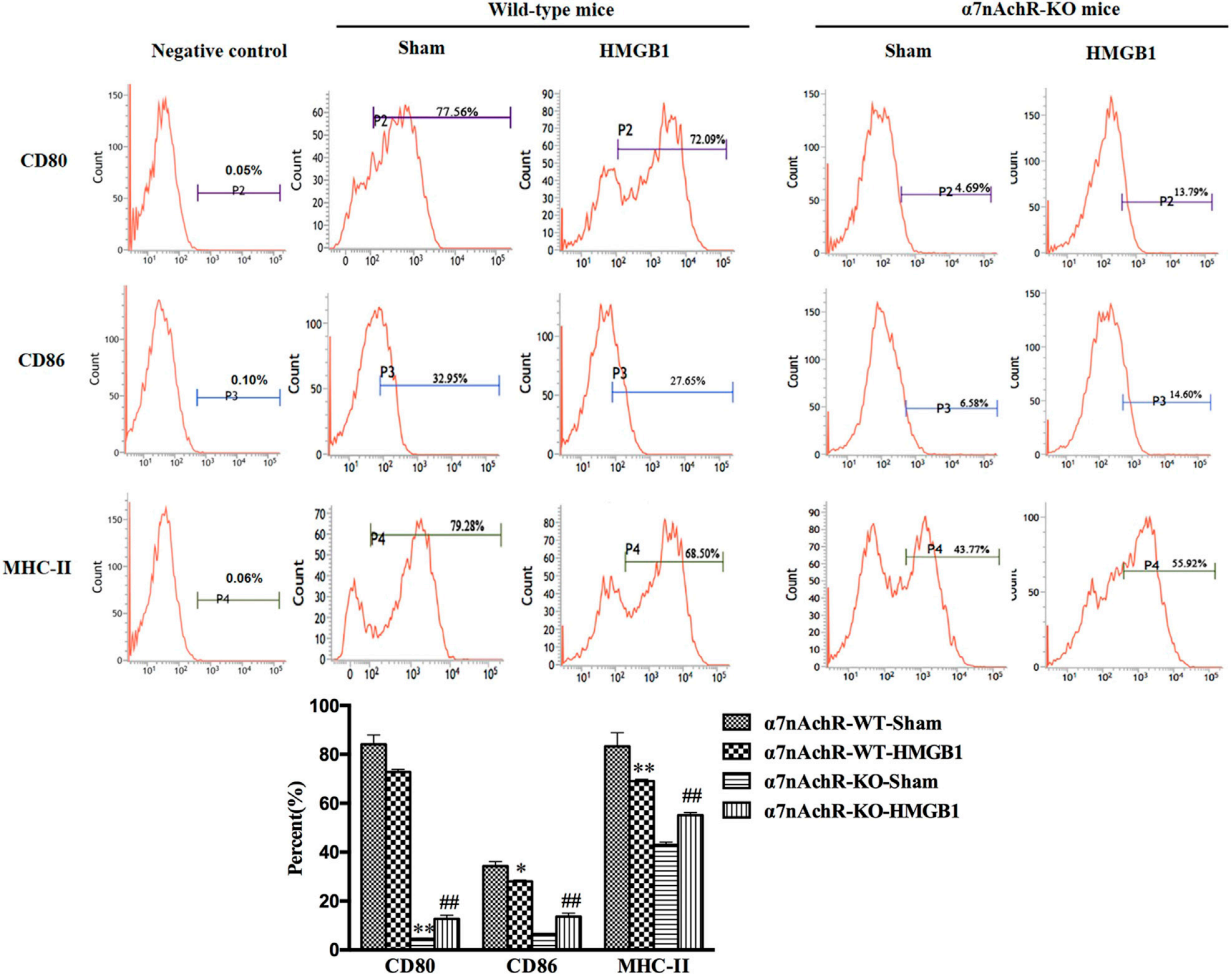
Blockade of α7nAchR abated the impact of brain HMGB1 on functional inhibition of splenic DCs. The expression of costimulatory molecules in DCs, including CD80, CD86, and MHC-II, was assessed by flow cytometry. The statistical results are presented to indicate these phenotypic changes (*n* = 6, vs*.* the α7nAchR-WT-sham group: ^*^ *p* < 0.05, **p < 0.01; vs*.* the α7nAchR-KO-sham group: ^##^ *p* < 0.01).

**FIGURE 10:**
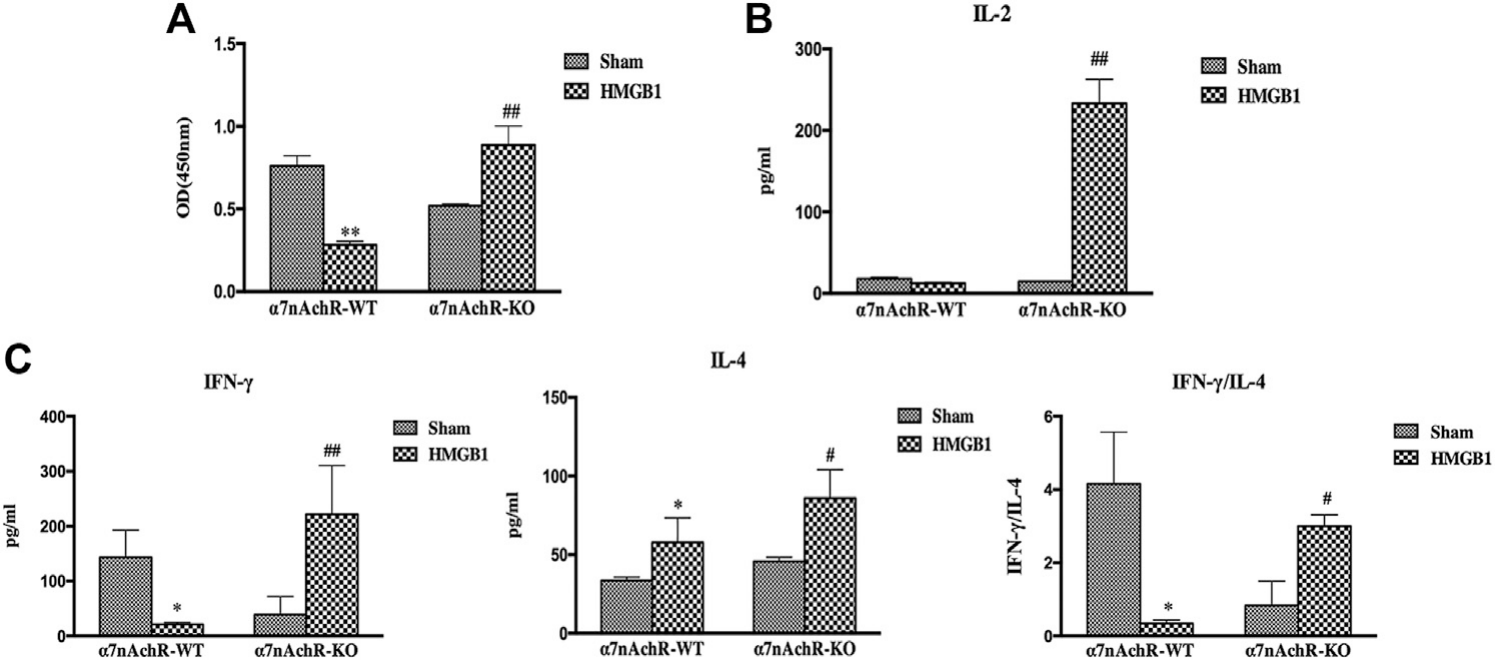
The effect of brain HMGB1 on splenic DC priming of T cells in mice with α7nAchR gene knockdown. **(A)** The proliferation of CD4^+^ T cells was measured by the CCK-8 assay, and quantified by an ELISA plate reader at 450 nm. **(B, C)** The levels of IL-2, IFN-γ, and IL-4 in coculture supernatants were determined by ELISA kits, and the ratio of IFN-γ/IL-4 was determined to reflect the polarization of CD4^+^ T cells (*n* = 6, vs*.* the α7nAchR-WT-sham group: ^*^ *p* < 0.05, **p < 0.01; vs*.* the α7nAchR-KO-sham group: ^#^ *p* < 0.05, ^##^ *p* < 0.01).

α7nAchR has been identified as the major receptor of CAP, which is extensively expressed in multiple immune cells (Wang et al., 2003). We found that α7nAchR-KO mice exhibited increased expression of CD80, CD86, and MHC-II in response to cerebral HMGB1 challenge, which showed the opposite trend when compared to that of wild-type (WT) mice (Figure 9). The priming activity of DCs for T cells in α7nAchR-KO mice was also restored when compared with those in WT mice, as evidenced by improved proliferation and increased IL-2 release under exposure with ICV injection of HMGB1 **(** Figures 10A,B). Furthermore, T cells also showed Th1 polarization when cocultured with DCs from α7nAchR-KO mice in response to ICV injection of HMGB1, as the ratio of IFN-γ to IL-4 was higher than that in the sham group **(** Figure 10C).

## Discussion

Extracellular HMGB1 is identified as a late inflammatory mediator that drives uncontrolled inflammatory response and is considered a potential therapeutic target for sepsis (Gentile and Moldawer, 2014). It has been documented that serum HMGB1 was significantly increased during sepsis and was responsible for multiple organ dysfunction and lethality in septic patients (Sun et al., 2015). Downregulating the release or activity of HMGB1 showed protection against multiple organ injury, along with significant improvements in the survival of septic animals (Pahuja et al., 2008; Gentile and Moldawer, 2014). HMGB1 was reportedly responsible for memory impairment in naïve mice, and the administration of anti-HMGB1 antibodies was beneficial for surviving animals by preventing cognitive decline (Chavan et al., 2012). We have previously confirmed that increased level of cerebral HMGB1 is a main cause of sepsis-induced brain injury. Early administration of BoxA, significantly attenuated dysfunction of central nervous system and reversed cognitive impairment, suggesting that neuroinflammation might be the major cause of brain injury in sepsis (Pahuja et al., 2008). Neuroinflammation has been documented as a critical threat to neuronal cells by inducing massive apoptosis (Pfister et al., 2008). Overproduction of inflammatory mediators is one of the major causes driving the exaggerated inflammatory microenvironment in the brain (Pytel and Alexander, 2009). Furthermore, uncontrolled local inflammation in the central nervous system might be unfavorable for the neuroendocrine immune networks in septic conditions and can lead to a vicious cycle of immunomodulation if without prompt interference.

In the present study, we found that increased level of brain HMGB1 was related to immune dysfunction of splenic DCs in sepsis, as shown by suppressed expression of surface molecules and disturbed priming activity for T cells. Antagonism of brain HMGB1 reversed the suppressed expressions of DC surface molecules, including CD80, CD86, and MHC-II, and improved T cell proliferation, cytokine secretion as well as the homeostasis of Th1/Th2 differentiation, suggesting that cerebral HMGB1 might also be responsible for sepsis-induced dysfunction of peripheral DCs. The anti-inflammatory phenotypes, including CD206 and CD163, were also evaluated and showed significant increases under persistent sepsis exposure. ICV injection of BoxA significantly decreased the CD206 expression but showed no influence on the level of CD163, hinting the partial effects of cerebral HMGB1 on the differentiation of DCs. The brain HMGB1 stimulation was capable of inhibiting activation of DCs and inducing differentiation of DCs into anti-inflammatory phenotypes, which further augmented the progression of sepsis-induced immunosuppression. It was noteworthy that the immune function of splenic DC showed a marked contrast between 24 vs. 48 h after the induction of sepsis, which might be responsible for the divergent effects of ICV injecting BoxA. The different performances of DC function were mainly due to the nature of septic animal model by CLP, a currently accepted golden standard for reproducing clinical sepsis (Rittirsch et al., 2009). The splenic DC presented with different functional status after exposed by CLP surgery for different time points, such as early initiation and later depression, which was also determined by the severity of sepsis (Wu et al., 2017). In this study, the splenic DC was found with activation at 24 h but significant suppression at 48 h post CLP surgery. The ICV injection of BoxA showed no significant influence in DC function at sepsis 24 h, while reversed its inhibited activation at 48 h after sepsis induction, suggesting that persistent release of brain HMGB1 could be detrimental to the immune response of DCs. We further performed ICV injection of HMGB1 and found that the function of DC was inhibited at 48 h after HMGB1 stimulation, which was favorable for understanding the relationship between elevated brain HMGB1 and disturbed immune response of peripheral DCs. These data provided novel insight into the role of cerebral HMGB1 on sepsis-induced immunosuppression, which might be due to the collapse of neuro-endocrine immune networks. The neuro-endocrine immune networks have been well established in various diseases by regulating immune and inflammatory responses (Yang et al., 2017). The CAP is well documented as a potent anti-inflammatory mechanism and reportedly benefits septic animals by ameliorating local and systemic inflammatory responses (Song et al., 2008). Dysfunction of the CAP, either hypo- or hyperactivity, however, has been reported to jeopardize the homeostasis of host immune system. An invalid CAP response due to cholinergic deficiency in the brain, vagotomy or blockade of α7nAchR, contributes to excessive production of inflammatory cytokines during sepsis (Wang et al., 2009). Hyperactivation of the vagus nerve is considered the major cause of immune paralysis in traumatic brain injury (Kox et al., 2008). Current knowledge well addressed the impacts and mechanism(s) of CAP in controlling excessive inflammation and alleviating multiple organ injury under septic exposure, followed by multiple potential drug targets in pre-clinical studies. However, the precise mechanism covering the functional changes of CAP and the interaction between inflammatory mediators and cholinergic system remained unclarified. We found that the brain cholinergic system showed marked activation at 48 h after CLP surgery, which was reversed by ICV injection of HMGB1 inhibitors. The levels of ChAT and AchE in the brain tissues were quantified and used as indicators for the activity of the brain cholinergic system. The expressions of ChAT in different areas of brain tissues were significantly increased at 48 h after CLP surgery, along with suppressed AchE levels, indicating significant activation of the brain cholinergic system. The levels of AchE in both serum and splenic samples showed the same tendency with brain tissues, indicating the systematic activation of cholinergic system. Given roles of ChAT and AchE in the synthesis and degradation of Ach respectively, the different performance of ChAT and AchE in response to sepsis suggested the enhanced activation of cholinergic system by increasing the production but decreasing the degradation of Ach.

We also observed a similar trend of enhanced cholinergic activity after ICV injection of HMGB1, suggesting that cerebral HMGB1 inhibited the function of splenic DCs mainly by inducing hyperactivation of the CAP. However, contradictory effects were found in α7nAchR-KO mice when compared with WT mice. In our study, the surface molecules of splenic DCs in α7nAchR-KO mice showed upregulated expressions in response to cerebral HMGB1, along with enhanced priming activity for T cells. These discrepant results between WT mice and α7nAchR-KO mice might result from compensatory mechanisms (Salamone et al., 2011). In addition to α7nAchR, DCs have also been shown to express multiple muscarinic receptors, including M3, M4, and M5, which exhibit totally different effects (Salamone et al., 2011). Activation of muscarinic receptors results in the phenotypic activation of DCs and secretion of pro-inflammatory cytokines (Pytel and Alexander, 2009). DCs exhibited downregulated HLA-DR expression, inhibited IL-12 production and incompetent T cell priming when blocking the activation of muscarinic receptors (Pytel and Alexander, 2009). Therefore, the enhanced activation of splenic DC with α7nAchR-KO might be due to the extensive activation of muscarinic receptors by increased production of acetylcholine in response to brain HMGB1. However, the specific mechanism remained to be clarified covering the distinct receptor and intracellular pathways for the compensatory effects with α7nAchR deficient.

The present study provided novel insights into the potential role of brain HMGB1 in modulating the peripheral immune response by inducing hyperactivation of the cholinergic system. These findings also promoted the understanding of sepsis-induced immunosuppression underlying the activity of neuroendocrine immune networks. Furthermore, current study highlighted the importance of early recognition and treatment of sepsis-induced brain injury, as this condition might become a vicious cycle for incompetent immune modulation. However, some limitations should be noted when interpreting these findings: Firstly, this study only described the effects of cerebral HMGB1 on the immune function of splenic DCs and its relationship with cholinergic system. However, the specific mechanisms, including the distinct pathway for immunosuppressive effects of cerebral HMGB1 and activation of cholinergic system, remain to be clarified in the future studies. Secondly, the mechanism of which HMGB1 upregulates the expression of ChAT but downregulates AchE expression also need further study. Thirdly, other proinflammatory cytokines and neuromodulatory pathways are also deserved to be investigated concerning their effects on peripheral immune response. Fourthly, given the critical involvement of the cholinergic system, we only provided evidence of its functional changes and impacts on the activation of DCs in the context of α7nAchR gene knockdown. Mice with conditional α7nAchR knockout, either in brain tissues or specific DCs, will provide more explicit information. Finally, the specific mechanism involved in restoring the DC functions after ICV injection of HMGB1 in α7nAchR-KO mice should be further explored.

## Conclusion

In the present study, we provided novel insights into the effects of cerebral HMGB1 on the immune function of splenic DCs under septic challenge. Increased release of brain HMGB1 was responsible for the abnormal response of DCs under septic conditions, which was partly related to the hyperactivation of the brain cholinergic system. Antagonism of brain HMGB1 was beneficial for the functional homeostasis of the CAP, and further showed protective effects on the immune response of peripheral DCs.

## Data Availability

The original contributions presented in the study are included in the article/supplementary material, further inquiries can be directed to the corresponding author.

## Abbreviations

Ach, acetylcholine; AchE, acetylcholinesterase; CAP, cholinergic anti-inflammatory pathway (CAP); CCK8, cell counting kit-8; ChAT, choline acetyltransferase; CLP, cecal ligation and puncture; DCs, dendritic cells; HMGB1, high mobility group box-1 protein; ICV, intracerebroventricular; IFN-γ, interferon-γ; IL-1β, interleukin-1β; MHC-II, major histocompatibility complex-II; PBS, phosphate buffer saline; TNF-α, tumor necrosis factor-α; α7nAchR, α7 nicotinic acetylcholine receptor.
